# Mechanisms of Beta-Blocker Induced Psoriasis, and Psoriasis De Novo at the Cellular Level

**DOI:** 10.7759/cureus.8964

**Published:** 2020-07-02

**Authors:** Vanessa M Awad, Sirisha Sakhamuru, Srikala Kambampati, Shehnaz Wasim, Bilal Haider Malik

**Affiliations:** 1 Internal Medicine/Family Medicine, California Institute of Behavioral Neurosciences and Psychology, Fairfield, USA; 2 Internal Medicine, California Institute of Behavioral Neurosciences and Psychology, Fairfield , USA; 3 Internal Medicine, California Institute of Behavioral Neurosciences and Psychology, Fairfield, USA

**Keywords:** beta-blocker side effects, beta-blocker induced psoriasis, drug-induced psoriasis, beta-blockade, psoriasis/drug therapy, calcium effects on keratinocytes, mechanism of beta-blocker psoriasis

## Abstract

Beta-blockers are a commonly prescribed medication, but the increase in use goes hand in hand with increasing side effects; one of particular interest lately has been its dermatological reactions. Although rare, beta-blockers can exacerbate pre-existing psoriasis and also cause de novo psoriasis in patients naïve to the disease. The mechanism by which this occurs is still unclear, although numerous articles have been published throughout the years as to how this unusual effect takes place. The most common mechanism suggests that beta-blockers cause intracellular changes in calcium, affecting both keratinocyte proliferation and granulocyte function via decreased cyclic adenosine monophosphate (cAMP) levels. Several inflammatory mediators are known to play a role, as well as reduced expression and desensitization of the beta-adrenergic receptor itself. We discuss these posed pathways in-depth and how each contributes to the worsening or formation of new psoriasis. With this knowledge, future physicians may be more mindful of this side effect should it occur, and why they occur, to better manage our patients on this widely used medication.

## Introduction and background

Beta-blockers: cardiac jacks of all trades

Beta-blockers are a very commonly used drug for the treatment and prevention of a vast amount of diseases; these can be both cardiac and non-cardiac. In the list of the most prescribed medications seen in the United States, carvedilol came 26th, and propranolol came 63rd [1,2]. The most common cardiac disorders for their use are coronary artery disease, heart failure, arrhythmias, and hypertension. Outside of the cardiovascular system, beta-blockers are also used for essential tremor, migraine, esophageal varices prophylaxis, thyrotoxicosis, and anxiety [3]. With its use, however, comes its side effects, such as fatigue, dizziness, gastrointestinal upset, and sexual dysfunction. But a dermatological one, in particular, has been gaining attention as of late. Although the mechanism is not fully understood yet, the use of beta-blockers is known to exacerbate pre-existing psoriasis, as well as precipitating it de novo [4,5]. The first scenario of exacerbation of psoriasis is defined as the cessation of the progression upon withdrawal of the agent in either pre-existing lesions or new lesions in uninvolved skin. De novo psoriasis is described as the occurrence of the disease in those with no personal or family history of the said disease [6].

Psoriasis is notoriously known to be associated with numerous drug use and its exacerbations, including de novo psoriasis in patients with no previous history of psoriasis. This drug-disease association has been widely assessed and documented through clinical trials, case reports, and literature reviews [7]. We will be discussing the association between beta-blocker drug use, and the exacerbation of pre-existing psoriasis, and de novo psoriasis. Both the cardioselective and non-cardioselective beta-blockers are known to cause such side effects, having practolol being the most frequently reported drug to do so [8,9]. Although it is now no longer used due to its unfavorable side effect profile, other and more effective beta-blockers are still found to cause such side effects regardless of the route of administration, whether it be oral, intravenous, or ophthalmologic [9].

This literature review will explore the different posed mechanisms by which this exacerbation of psoriasis and de novo psoriasis occurs at the cellular levels by beta-blocking agents (Table 1). This review can help clinicians become aware of this rare, yet commonly documented side effect of such a frequently prescribed drug. Though many hypotheses are posed, and although no consensus is reached, we will review the most frequently documented theories to date.

**Table 1:** Common hypotheses for mechanism of beta blockade at cellular levels The table depicts the different hypotheses collected among 38 papers, their central premise, the number of articles that overlapped in hypotheses, and the year of publication range from these studies. Abbreviation: cAMP, cyclic adenosine monophosphate

Hypothesis	Number of studies collected	Studies overlapping in hypothesis	Year of Publication Range
cAMP in Keratinocytes	21	13	1972-2019
cAMP in Granulocytes	8	8	1973-2014
Inflammatory Mediators	5	2	1993-2019
Other hypotheses	4	3	1975-2004

## Review

Beta-adrenergic receptors are found throughout the body, ranging from type 1 to type 3. From previous research, we now know that the beta-adrenergic receptors found of the surface of keratinocytes are of subtype 2. This subtype is known to be the start point of the activation or the blocking cascade. [9].

Basal cells contain the highest concentration of subtype 2 receptor, and gradually decrease moving toward the stratum corneum. For intracellular calcium, however, basal cells contain the lowest levels, and this concentration increases moving towards the stratum corneum, which keeps in line with the differentiation of keratinocytes [10].

 

Cyclic adenosine monophosphate

Keratinocytes

Usually, when the beta-adrenergic subtype 2 receptors become activated on keratinocytes, this activates adenyl cyclase, the enzyme that will form cyclic adenosine monophosphate (cAMP). As the intracellular levels of cAMP increase, this causes intracellular calcium to increase. Calcium increase in cells thereby stimulates proteins responsible for the regulation of differentiation of cells and inhibition of keratinocyte proliferation [9,11,12].

With beta-blocker use, these receptors and adenyl cyclase are no longer activated, decreasing cAMP, and intracellular calcium levels. This decrease causes a detrimental cascade, causing dysregulation of differentiation and promoting keratinocyte proliferation. It’s posed that intracellular calcium changes cause modifications in the cells' filament network and specific keratinocyte granules, which therein causes changes to cellular differentiation as well [13].

**Figure 1:**
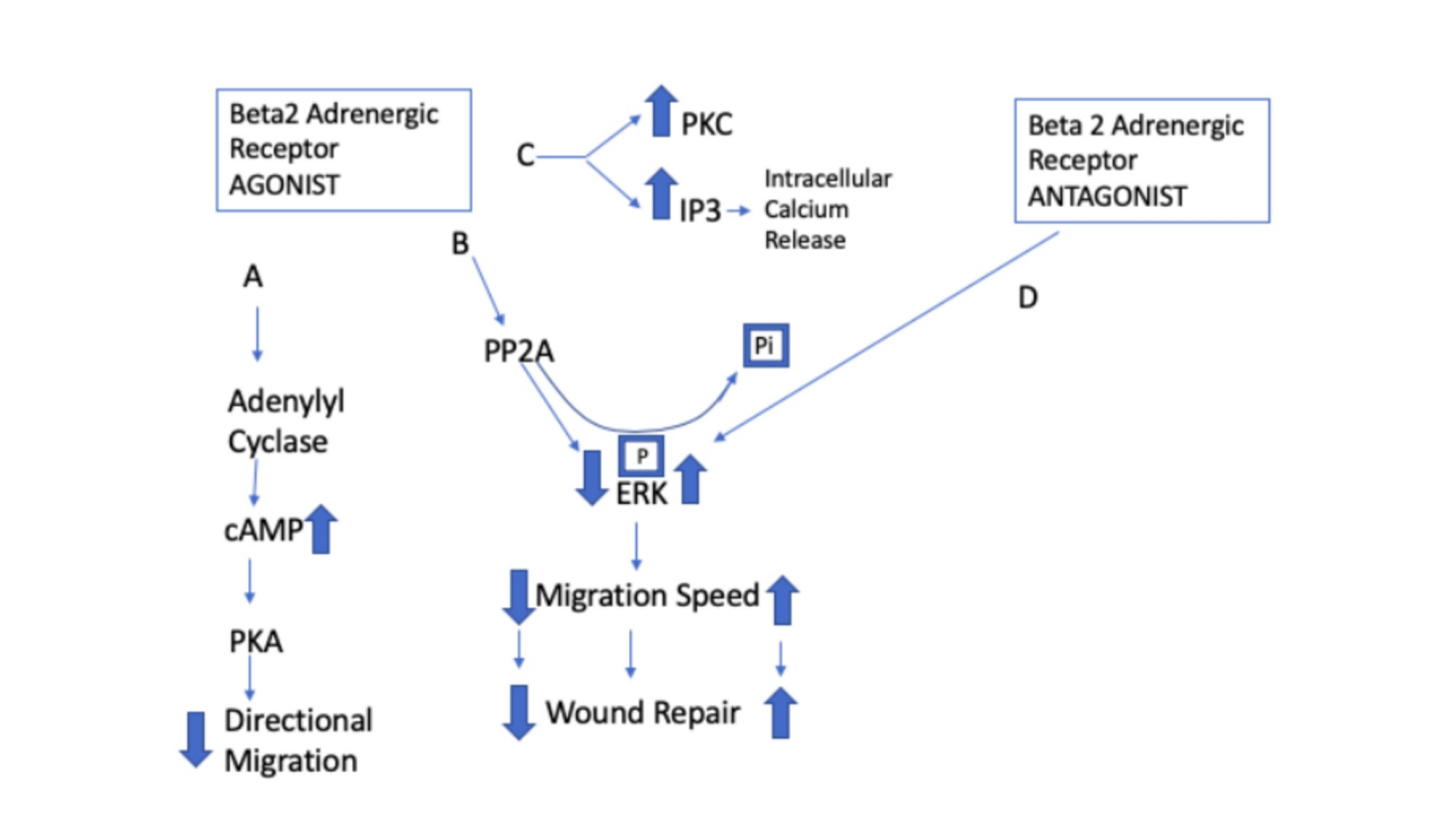
Stimulation and blockade of keratinocyte signalling pathways of beta2 adrenergic receptors PKA, protein kinase A; cAMP, cyclic adenosine monophosphate; PP2A, serine/threonine phosphatase. ERK, extracellular signal-related kinase; IP3, inositol- 1,4,5- triphosphate; PKC, protein kinase C. Pathways A, B, C, and D depict the various signalling pathways concerning keratinocyte receptor stimulation and blockade [17]

As seen in Figure 1, the effects of calcium on keratinocyte proliferation can be brought about via multiple pathways. A decrease in cAMP may also facilitate the breakdown of glycogen in keratinocytes, causing its accumulation intracellularly. Knowing the exact relationship between cAMP, decreased calcium levels, and the glycogen pathway is itself unknown. In a study by Voorhees et al., they noted that a buildup in glycogen may also have an effect on psoriatic eruptions caused by the beta-blocking cascade [14,15]. The compound effects of these pathways reveal an increase in epidermal cell turnover. With the continued use of beta-blockade, this cycle continues, causing more exacerbation of psoriasis in both patients with the disease and those without it. This hypothesis has been the most frequently documented and published; therefore, it is the most probable one as of now [16].

 

Granulocytes

Beta 2 subtype receptors are not only seen on keratinocytes but at the membranes of granulocyte cells as well. This effect of calcium in these cells also plays a role in the inflammatory process of psoriasis [18,19].

As the receptor becomes blocked, the cAMP levels fall, and it may correlate with increasing granulocyte motility; it can also lead to inducing DNA synthesis and mitogenesis. This increase in motility thereby increases microvillous formation within these cells. The decrease in cAMP can also signal a green light for neutrophils, macrophages, and lymphocytes to release their lysosomal enzymes, causing an abundance of proteolytic enzymes in the skin, aggravating the inflammatory mechanisms [18,19]. These events are also thought to be culprits of hyperproliferation of keratinocytes to bring about these psoriasiform changes, all due to decreased cAMP levels [19,20].

 

Inflammatory mediators

The body's inflammatory responses revolve around the reaction and release of cytokines and interleukins. Some studies resulted in demonstrating if there are effects of beta-blockade on cytokine and interleukin activity. In one study, the results show that the use of propranolol enhances higher gene expression of Interleukin-16 (IL-16) and other growth factors, having the ability to control, or in this case exacerbate, the immunologic reactions seen in psoriasis [21]. Another study found that propranolol's effects on the skin's macrophagic cells induce reactive oxygen species, which is essential for the release of IL-23 [22]. IL- 23 is described as one of the most important cytokines in the inflammatory process of psoriasis, as it is necessary for activating and maintaining Th17. Th17 thereby secretes more pro-inflammatory cytokines, mainly IL-17, IL-22, IL-21, and tumor necrosis factor-alpha (TNF- alpha) [23-25].

The Koebner Phenomenon, defined as trauma-induced psoriasiform lesions particularly in those who are psoriasis naïve, is also described to bring about changes in the inflammatory process [26]. This inflammation is ultimately caused by IL-17 [27]. Trauma, either physical or chemical, induces mast cell-derived IL-17, and at the end of its signaling pathway, IL-17 provokes the formation of new psoriatic lesions [27]. Combining this inflammatory process with beta-blocker use can cause additive effects of promoting de novo psoriasis.

It’s also been shown that the macrophage migration inhibition factor (MIF), a lymphokine representative of cellular immunity, plays a role in propranolol induced psoriasis. In a study by Halevy et al., 33 patients, consisting of both psoriasis affected and psoriasis naïve subjects, were shown to have a higher MIF response when treated with a beta-blocking agent, than the control subjects (45.4% and 2.7%) [28]. By these results, we may deduce that an immunologic mechanism can play a role in these psoriatic eruptions in patients affected by psoriasis and those naïve to the disease [28].

Other hypotheses

Not only do the beta-adrenergic receptors affect the cAMP pathway upon blockade, but they have been observed to be fewer in number in those with pre-existing psoriasis [29].

As the decreased expression of these receptors rises, the cAMP pathway exhibits a reduced response, rendering this pathway ineffective and causing decreased intracellular calcium and its cellular effects. Also, the reverse transcriptase-polymerase chain reaction (RT-PCT) shows decreased mRNA production of beta 2 adrenergic receptors, reducing its expression [30].

Other data has found that polymorphisms involving amino acid substitutions in the beta 2 adrenergic receptor genes can alter its properties, and therefore causing desensitization of these receptors [31]. In a study involving 50 subjects, substitutions were found at positions 16 and 27 of the receptor gene. This lead to an arginine substitution that was found to be in higher expression in the psoriatic patients compared to the controls group [31,32]. Combining the decreased expression of Beta 2 receptors and decreased sensitization, these factors are highly likely to contribute to these effects of beta-blockade and the results of this cascade.

Despite these many hypotheses, to fully comprehend these pathways, future physicians must continue this research in order to better manage patients suffering from the disease.

Limitations

Quality assessment was not done in this traditional review.

## Conclusions

This literature review aims to showcase and elaborate on the most commonly hypothesized mechanisms by which beta-blockade exacerbates psoriasis, and psoriasis de novo. The cAMP pathways and cascades proved to be the most frequent of hypotheses, followed by inflammatory mediators, the hallmark of psoriasis. Additionally, few studies have shown that the beta 2 adrenergic receptor itself, either decreased production or decreased sensitization, were also discovered. A challenge to our medical community includes finding other treatments for psoriasis that would induce the expression of beta-adrenergic receptors on keratinocytes or ways to hinder this mechanism without changing our patients' drug regimens. With this solution, we may not only provide relief for patients affected by psoriasis but leave patients on beta-blockers for those diseases that have been shown marked improvement with beta-blocker use.
